# Targeting Notch-1 positive acute leukemia cells by novel fucose-bound liposomes carrying daunorubicin

**DOI:** 10.18632/oncotarget.9558

**Published:** 2016-05-23

**Authors:** Michihiro Ono, Rishu Takimoto, Takahiro Osuga, Yutaka Okagawa, Masahiro Hirakawa, Makoto Yoshida, Yohei Arihara, Naoki Uemura, Naoki Hayasaka, Shogo Miura, Teppei Matsuno, Fumito Tamura, Yasushi Sato, Tsutomu Sato, Satoshi Iyama, Koji Miyanishi, Kohichi Takada, Masayoshi Kobune, Junji Kato

**Affiliations:** ^1^ Department of Medical Oncology and Hematology, Sapporo Medical University School of Medicine, Sapporo, Japan; ^2^ Division of Clinical Oncology, Sapporo Medical University Graduate School of Medicine, Sapporo, Japan; ^3^ Division of Molecular Oncology, Sapporo Medical University Graduate School of Medicine, Sapporo, Japan

**Keywords:** AML, Notch-1, targeting, liposome, L-fucose

## Abstract

Complete remission by induction therapy in acute myelogenous leukemia (AML) can be achieved due to improvements in supportive and optimized therapy. However, more than 20% of patients will still need to undergo salvage therapy, and most will have a poor prognosis. Determining the specificity of drugs to leukemia cells is important since this will maximize the dose of chemotherapeutic agents that can be administered to AML patients. In turn, this would be expected to lead to reduced drug toxicity and its increased efficacy. We targeted Notch-1 positive AML cells utilizing fucose-bound liposomes, since activation of Notch-1 is required for O-fucosylation. Herein, we report that intravenously injected, L-fucose-bound liposomes containing daunorubicin can be successfully delivered to AML cells that express fucosylated antigens. This resulted in efficient tumor growth inhibition in tumor-bearing mice and decreased proliferation of AML patient-derived leukemia cells. Thus, biological targeting by fucose-bound liposomes that takes advantage of the intrinsic characteristics of AML cells could be a promising new strategy for Notch-1 positive-AML treatment.

## INTRODUCTION

Approximately sixty percent of patients with acute myelogenous leukemia (AML) will achieve complete remission by induction therapy due to improvements in supportive, and increasingly optimized, therapy [1]. However, more than 20% of patients will still need to undergo salvage therapy, with most having a poor prognosis [2]. Based on such previous reports, determining the specificity of drugs to leukemia cells is important to improve a considerable proportion of AML patients' prognoses, since minimizing the dose of administered chemotherapeutic agents would be expected to lead to reduced toxicity and increased efficacy.

Notch-1 is a homolog of the translocation-associated Notch homologue (TAN-1) discovered in the t(7;9)(q34;q34) translocation in some T-cell acute lymphoblastic leukemia patients (T-ALL) [3]; its ligands are modified by a protein O-fucosyltransferase 1 (POFUT1) that attaches L-fucose to a serine or threonine within EGF domains [4, 5]. High expression of Notch-1 has been reported to be a predictive marker for a poor prognosis in AML patients [6] and is thus proposed as a therapeutic target in hematological malignancy [7, 8]. However, targeting Notch-1 by using an activator such as γ-secretase, or by the deletion of *POFUT1*, has been shown to result in gastrointestinal toxicity [9]. Therefore, directly targeting Notch-1 or the specific delivery of γ-secretase to Notch-positive leukemia cells is necessary to improve treatment strategies.

L-fucose is a monosaccharide that is a common component of many N- and O-linked glycans and glycolipids produced by mammalian cells [10]. As reported previously [11], we focused on the specific biological characteristics of metastatic pancreatic cancer, especially fucosylated antigens such as sialyl Lewis X (SLX) antigen and carbohydrate antigen-19-9 (CA19-9), which are found in the sera and tumor tissues of patients [12, 13] and are used as tumor markers in detecting cancer and in the evaluation of treatment efficacy. Furthermore, enhanced expression of fucosyltransferases (FUTs) has been reported in various cancers. FUTs are key enzymes accelerating malignant transformation through the fucosylation of different sialylated precursors. It has been reported that enhanced activity of FUT3 is associated with the increased metastatic potential of colon and pancreatic adenocarcinoma cells [14], suggesting that fucosylation may play an important role in disease progression. These observations indicate a high requirement for L-fucose by various cancer cells [15]. Indeed, we, together with others, have reported that enhanced FUT activity facilitated the metastatic potential of colorectal cancer cells, thus highlighting this as a therapeutic target for cancer therapy [16].

The siglecs (sialic acid-binding immunoglobulin superfamily lectins) are immunoglobulin superfamily members that recognize sialylated ligands [17]. Most prior studies of siglec specificities have focused on α 2–3- and α 2–6-sialyllactos (amin)es, and on one or two of the siglecs at a time. Of the various existing siglecs, siglec-3 (CD33), which is expressed in AML patients, has been reported to be affected markedly by the presence of an α1–3-linked fucose. To target AML cells directly, therefore, CD33 seems to be a promising molecule.

In this study, we targeted Notch-1 positive AML cells by utilizing fucose-bound liposomes, since activation of Notch-1 is required for O-fucosylation by POFUT. Herein we report that intravenously injected L-fucose-bound liposomes containing daunorubicin can be successfully delivered to AML cells that express fucosylated antigens. This resulted in efficient tumor growth inhibition in tumor-bearing mice. Thus, biological targeting by fucose-bound liposomes utilizing characteristics intrinsic to AML cells could be a promising new strategy for AML treatment.

## RESULTS

### Expression of CD33 and Notch-1 in leukemia cell lines

**Figure 1:**
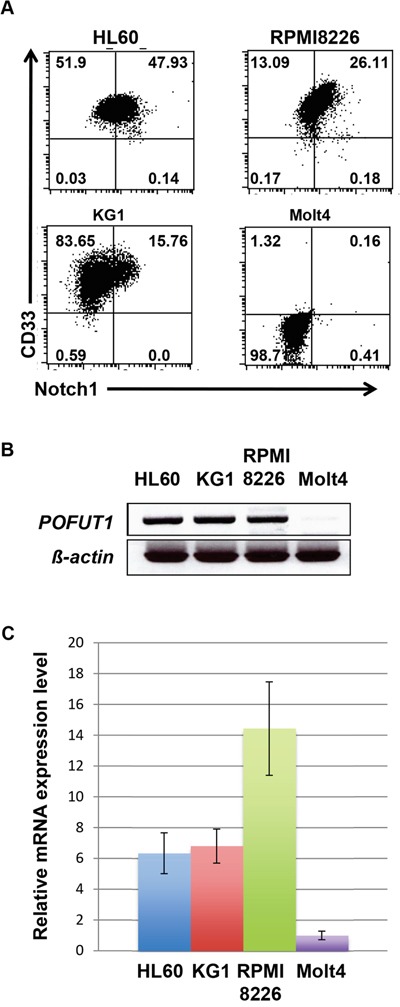
Expression of POFUT1 in Notch1 positive cell lines **A.** Flow cytometric analysis of HL60 and CD33 expression in leukemia cell lines HL60, RPM8226, KG1 and Molt4. Expression of POFUT1 mRNA in leukemia cells lines examined by semi-quantitative **B.** and quantitative **C.** PCR. These experiments were performed three times.

We first analyzed the expression of CD33 and Notch-1 in various leukemia cell lines. As shown in Figure 1A, the expression of CD33 was observed in all cell lines except for Molt4. It should be noted that RPMI8226 cells, which were derived from a multiple myeloma, expressed an appreciable amount of CD33 as previously reported [18]. Notch-1 expression was also examined, being found in HL60, RPMI8226, and KG1 cells, but not in Molt4 cells. We then analyzed the mRNA expression of POFUT1 in various leukemia cells line (Figure 1B, 1C, Supplementary Figure 1) and found that this directly correlated with Notch-1 expression. However, we did not observe a relationship between other FUTs and Notch-1 expression (Supplementary Figure 1). Thus, the expression of CD33 and Notch-1 correlated in the same leukemia cell lines.

### Targeting Notch-1 positive cells by fucose-bound liposomes

We then investigated whether Notch-1 positive leukemia cells could be targeted by L-fucose-bound liposomes. As reported previously [11], aminated L-fucose was cross-linked via 3,3′-dithiobis[sulfosuccinimidylpropionate] (DTSSP) to liposomes prepared by a modified cholate dialysis method to achieve a final concentration of 25 (F25) or 50 μg/ml (F50). Furthermore, bis(sulfosuccinimidyl)suberate (BS^3^) and Tris were then coupled to hydrophilize the liposome surface, which normally prevents uptake by the reticuloendothelial system in the liver and spleen, as well as by macrophages and vascular endothelial cells, and can also prevent adsorption to opsonin proteins in plasma; consequently, systemic retention of liposomes is prolonged [19]. Almost all L-fucose-bound liposomes (Fuc-Liposomes) were spherical in shape and, in the case of FAM- and daunorubicin, were approximately 70 – 100 and 170 nm, respectively, in size (Supplementary Tables 1 and 2). The zeta-potential, representing the negative electric charge of the liposome surface, was below −40 mV (Supplementary Tables 1 and 2), which meant it was sufficiently hydrophilized for stealth functions.

**Figure 2:**
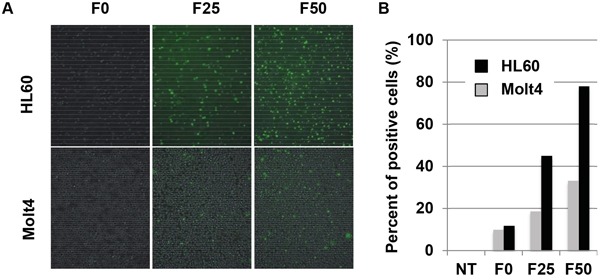
Introduction of Fuc-liposome-FAM into leukemia cell lines Fucose was crosslinked to liposomes at 0 (F0), 25 (F25) or 50 (F50) μg/ml. HL60 or Molt4 cells were incubated with F0-, F25-, and F50-liposomes-FAM, and after 30 min, cells were analysed by fluorescence microscopy **A.** or by flow cytometry after 2 h incubation **B.** Experiments were repeated at least three times. NT: no treatment.

Using these Fuc-Liposomes, we added FAM encapsulated L-fucose-liposomes (Fuc-Liposome-FAM) to Notch-1 positive or negative leukemia cell lines to confirm the specificity of delivery. As shown in Figure 2A, fluorescence microscopy revealed that F50-Fuc-Liposomes, but not F0-Fuc-Liposomes, effectively introduced FAM into the cytosol of HL60 cells. However, Molt4 cells, which do not express Notch-1, did not show uptake of FAM. Furthermore, flow cytometry analysis showed that after 2 h of incubation, F50-Fuc-Liposomes, but not F0-Fuc-Liposomes, effectively introduced FAM into the cytosol of HL60 cells that expressed Notch-1 (Figure 2B). Since we previously confirmed that uptake of L-fucose is mediated by its receptors [11], excess L-fucose decreased the efficiency of this process (data not shown), indicating that the introduction of FAM by Fuc-Liposomes is indeed L-fucose-dependent. Thus, Notch-1 positive leukemia cells were found to be targeted by L-fucose-bound liposomes.

### Effect of Fuc-Liposome-daunorubicin on the growth of leukemia cell lines

**Figure 3:**
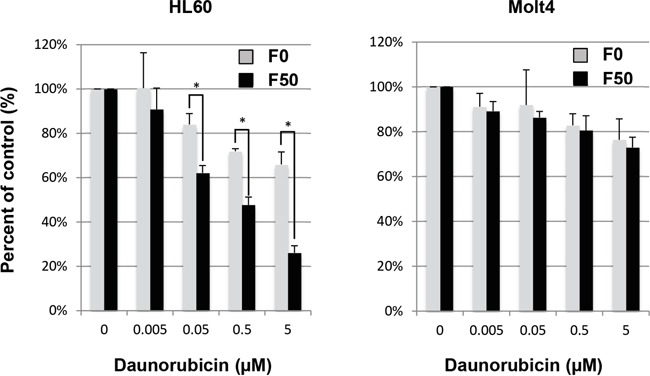
Effect of Fuc-liposome-daunorubicin on growth of cultured leukemia cell lines HL60 or Molt4 cells were incubated with F0 (no fucose) or F50 (50 μg/ml fucose) Fuc-liposomes carrying daunorubicin as indicated for 2 h, then washed with PBS and incubated for a further 72 h. Viable cells were calculated by BrdU assay as described in Materials and Methods. Cell growth was expressed as a percentage of untreated control. Experiments were performed in triplicate and repeated at least three times. *p < 0.05

We then encapsulated daunorubicin into Fuc-Liposomes. Fuc-Liposome-daunorubicin particles were approximately 170 nm in size, and the final concentration of daunorubicin was estimated at 1 mg/ml (Supplementary Table 2). This size of nanoparticle is postulated to allow penetration through the smallest capillary pores within the cancer vasculature by an enhanced permeability and retention (EPR) effect, but without breaching the blood-brain barrier [20]. The cytotoxicity of Fuc-Liposome-daunorubicin was tested using a BrdU cell proliferation assay (Figure 3). Cultured leukemia cells were exposed to Fuc-Liposome-daunorubicin or Liposome-daunorubicin for 2 h and then washed twice with phosphate-buffered saline to test the efficacy and specificity of daunorubicin transfer into Notch-1 positive cells. Because we observed the greatest cytotoxicity using F50-Liposome-daunorubicin (50 μg/mL fucose; Figure 3), we selected this condition for the following experiments. In Notch-1 positive cells (HL60, KG1), F50-Liposome-daunorubicin exerted more potent effects than control liposomes (F0-Liposome-daunorubicin), indicating fucose-dependent cytotoxicity (Figure 3). In addition to the effects on Notch-1 leukemia cell lines, the growth of other cancers, such as pancreatic and biliary cancer cell lines which produce CA19-9, were effectively suppressed by F50-Liposome-cisplatin, indicating the potential applicability of this nanoparticle technology for the treatment of different types of cancer (data not shown) [11]. Moreover, cytotoxicity was not seen in normal cells such as peripheral blood mononuclear cells, fibroblasts, human umbilical vein endothelial cells (HUVEC), or primary keratinocytes, probably due to their low requirement for L-fucose, as previously described [11]. Therefore, Fuc-Liposome-daunorubicin particles were found to inhibit the growth of Notch-1 positive cells *in vitro* in a fucose-dependent manner without affecting various normal cell types.

### Fuc-Liposomes carrying daunorubicin suppressed tumor growth and prolonged survival of mice in a xenograft model

**Figure 4:**
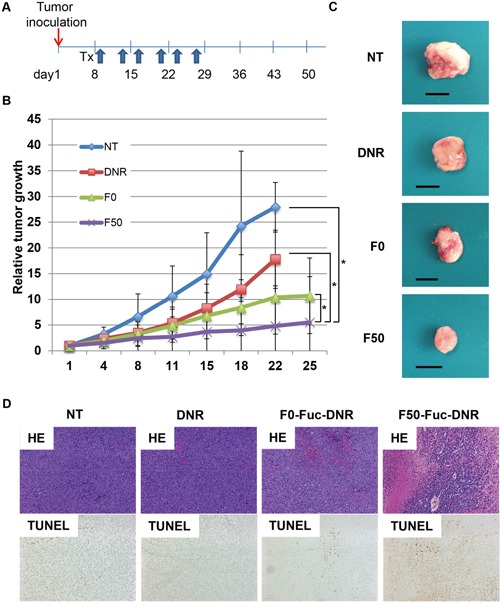
Fuc–liposomes carrying daunorubicin suppressed tumor growth in a xenograft model **A.** Treatment schedule for the HL60 xenograft model. Treatments were administered via tail vein injection to tumor-bearing mice twice a week for three weeks. **B.** Comparison of tumor growth suppression with daunorubicin, F0-liposome-daunorubicin (no fucose), or F50-liposome-daunorubicin (50 μg/mL fucose) in HL60-bearing mice with 1 mg/kg daunorubicin used for each treatment. At 1, 4, 8, 11, 15, 18, 22, and 25 days after transplantation, tumor volumes were measured. **C.** Tumor tissue was prepared on day 22 after treatment. **D.** Hematoxylin eosin (HE) staining (upper panel) and TUNEL staining (lower panel) are presented (x 400). n = 6 mice per group *P < 0.01 compared with no treatment (NT), daunorubicin (DNR), and F0.

In order to test the effects of Fuc-Liposome-daunorubicin on tumor growth *in vivo*, we developed a HL-60 xenograft model in mice. These animals were treated with Fuc-Liposome-daunorubicin twice a week for three weeks (Figure 4A). Tumor growth was significantly inhibited by treatment with F50-Liposome-daunorubicin compared with no treatment, F0-Liposome-daunorubicin, or daunorubicin alone in HL60-bearing mice, suggesting that F50-Fuc-Liposome delivered daunorubicin specifically and efficiently (Figure 4B; ***P* < 0.01,** 4C). In hematoxylin-eosin staining of tumor tissues, many viable tumor cells were observed in untreated mice (Figure 4D). In contrast, the number of tumor cells decreased in daunorubicin-treated and F0-Liposome-daunorubicin treated-mice as compared with untreated mice. However, in mice treated with F50-Liposome-daunorubicin, tumor cells almost completely disappeared. TUNEL staining revealed the presence of greater numbers of apoptotic cells in tumors treated with F50-Liposome-daunorubicin than in controls (Figure 4D), possibly due to the more marked accumulation of daunorubicin in tumor tissue.

No adverse effects, including body weight changes, attributable to the administration of either D-mannose or F50-Liposome-daunorubicin were observed during this study (data not shown). Thus, as with *in vitro* experiments, Fuc-Liposome-daunorubicin inhibited tumor growth *in vivo* in a HL-60 xenograft model in mice, possibly by an apoptotic mechanism.

### Fuc-Liposomes carrying daunorubicin suppressed Notch-1 positive AML cells from patients

**Figure 5:**
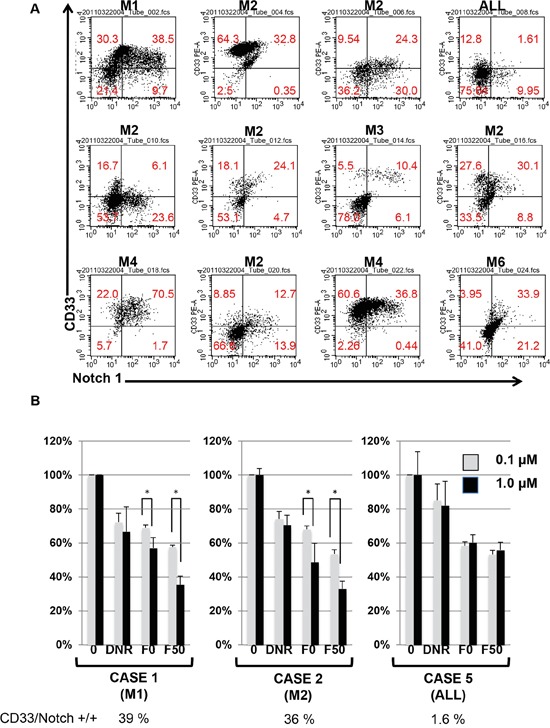
Effect of Fuc-liposome-daunorubicin on the growth of leukemia cells from patients **A.** Expression of CD33/Notch-1 in Acute leukemia patients' cells. Leukemia cells derived from patients were analysed by flow cytometry. AML patients (M1, M2, M3, M4, M5), ALL patient. **B.** Effect of Fuc-liposome-daunorubicin on the growth of leukemia cells derived from AML patients. AML patient-derived leukemia cells were incubated with Fuc-liposome-daunorubicin for 2 h and washed with PBS. After a further 72 h incubation, viable cells were measured by BrdU cell proliferation assay. AML (M1, M2) or ALL patients. Cell growth was expressed as a percentage of untreated control. The percentage of CD33/Notch-1 positive cells is demonstrated below. Experiments were performed in triplicate and repeated three times. Gray column and black column represented the data treated with 0.1 and 1.0 μM Fuc-liposome-daunorubicin, respectively. *p < 0.05.

We extended our observations to testing leukemia cells derived from AML patients. As shown in Figure 5 and Tables 1 and 2, 11 cases out of 12 AML patients were found to express Notch-1. Furthermore, CD33 expression by leukemia cells from patients also seemed to be compatible with Notch-1 expression. As shown in Figure 5A and Supplementary Figure 2, a correlation was observed between CD33 and Notch-1 expression at R = 0.534 (*P* < 0.001). However, clinical presentation, such as prognosis, sex, age, and remission rate did not correlate with Notch-1 expression (data not shown). We examined the effect of Fuc-Liposome-daunorubicin on leukemia patients' Notch-1 positive and negative cells. As shown in Figure 5B, F50-Liposomes containing daunorubicin effectively suppressed Notch-1 positive cells but not negative cells, indicating that fucose bound-liposomes specifically targeted Notch-1 positive leukemia cells isolated from patients. However, further investigation is required to verify the specificity and efficacy of F50-Liposomes containing daunorubicin. Thus, fucose-bound liposomes carrying daunorubicin suppressed Notch-1 positive, but not Notch-1 negative, AML cells from patients.

**Table 1:** Patient characteristics

No	Diagnosis	Gender	Age	Treatment
1	AML (M1)	M	81	BSC
2	AML (M2)	F	57	IDR/AraC
3	AML (M2)	M	66	IDR/AraC
4	ALL	F	71	CY/DNR/VCR/L-ASP/PSL
5	AML (M2)	M	64	IDR/AraC
6	AML (M2)	M	44	BMT
7	AML (M3)	M	49	
8	AML (M2)	M	66	IDR/AraC
9	AML(M4)	F	43	IDR/AraC
10	AML (M2)	F	21	BMT
11	AML(M4)	M	64	BMT
12	AML (M6)	M	64	BMT
13	AML (M2)	F	80	BSC

AML: acute myelogenous leukemia, ALL: acute lymphoblastic leukemia

BSC: best supportive care, IDR: idarubicin, AraC: Cytarabine, PSL: prednisolone, CY: cyclophosphamide, DNR: daunorubicin, VCR: vincristine, L-ASP: L-asparaginase, BMT: bone marrow transplantation

**Table 2:** Expression of Notch1 and/or CD33 in leukemia patients

No	Diagnosis	CD33 x Notch1	Notch1	CD33
1	AML (M1)	38.53	48.56	68.84
2	AML (M2)	32.83	33.64	97.13
3	AML (M2)	24.27	54.5	33.81
4	ALL	1.61	11.76	14.41
5	AML (M2)	16.68	29.8	22.75
6	AML (M2)	24.13	29.34	42.26
7	AML (M3)	10.42	16.91	15.93
8	AML (M2)	27.59	39.2	57.69
9	AML(M4)	70.54	72.51	92.54
10	AML (M2)	12.65	26.78	19.49
11	AML(M4)	36.75	37.46	97.3
12	AML (M6)	33.87	55.73	37.81
13	AML (M2)	7.65	15.44	37.55

## DISCUSSION

Presently, more than 20% of AML patients are required to undergo salvage therapy, with most having a poor prognosis [2]. Therefore, increasing the specificity of drugs to leukemia cells should lead to an improvement in the prognoses of a considerable proportion of AML patients, and minimizing the dose of chemotherapeutic agents administered would be expected to lead to their reduced toxicity and increased efficacy. The present study demonstrated that fucose-bound liposomes carrying daunorubicin could effectively target Notch-1/CD33 positive leukemia cells using the intrinsic requirement for L-fucose by such cells.

Notch-1 has previously been identified as a TAN-specific mutation in T-ALL patients [3]. Recent investigations have highlighted that Notch-1 shows a variety of roles in maintaining homeostasis in human cells and also plays an important role in carcinogenesis [5]. Such findings demonstrated that Notch-1 signaling maintained tumor growth, antiapoptotic signaling, epithelial-mesenchymal transition for cancer cell metastasis, angiogenesis, and cancer stem cell survival [5]. Our strategy to target cancer cells, which actively take up L-fucose to produce fucosylated proteins such as sialylated antigens and growth factors in response to Notch-1 activation, can be utilized for killing Notch-1-expressing cancer cells in the same manner [16]. As described in this report, our drug delivery system has the potential to specifically target cancer cells with reduced toxicity and high efficacy.

We did not find any adverse events when fuc-liposomes carrying an anti-cancer drug were administered twice a week to tumor-bearing mice, suggesting that its non-specific distribution to normal tissue was very low due to a high accumulation in tumors. The positive-targeting of cancer cells by a specific molecule using antibody-bound liposomes had been expected to be less toxic due to its specific distribution; however, this strategy presented several obstacles. Antigens targeted by modified liposomes are not specific to tumor cells, and may be competed with by antigens existing in the blood, resulting in damage to normal tissue or cells. Gemtuzumab ozogamicin (GO) was developed using this concept of directly targeting AML cells, and has been shown to be effective in relapsed AML patients [21]. However, remission rates have typically not exceeded 25% [22], indicating that targeting CD33 in the treatment of AML patients also requires improvements in strategy.

In regard to this issue, fucose-bound liposomes accumulated only in the liver; this distribution was eradicated by the administration of D-mannose, indicating that tissues other than liver do not actively take up L-fucose under normal conditions. In normal bone marrow cells, Notch-1 expression has been reported to be very low compared with leukemia cells [23, 24]. This phenomenon has also been confirmed in our previous report [11]. Thus, fucose-bound liposomes carrying anticancer drugs may be less toxic than any other drug delivery system already in clinical use.

It has been shown that cancer stem cells can self-renew symmetrically or can divide asymmetrically to produce daughter cells, which continue to proliferate to sustain tumor growth. Notch-1 has been demonstrated to play an important role in regulating cancer stem cells [25], suggesting that fucose-bound liposomes could also target these cells. Indeed, we found that pancreatic cancer stem-like cells expressing CD24/CD44 were targeted by L-fucose liposomes (data not shown). These results suggest that the majority of AML cells, including cancer stem cells, that exist during relapse or are resistant to conventional chemotherapy, may effectively be treated by L-fucose liposome therapy.

The Notch signaling pathway is also involved in the acquisition of drug resistance against chemotherapeutic agents, and in the activation of anti-apoptotic pathways in cancer cells. Molecules involved in these pathways have been shown to activate the PI3K-AKT axis and NFκB, as demonstrated by the use of a γ-secretase (GSI), and Notch-1 inhibitors in pancreatic, colorectal and breast cancers [26–28]. Thus, it might be expected that drug resistant clones, including Notch-expressing cells, may be effectively eradicated by a single administration of fucose-bound liposomes, resulting in early remission for patients with AML.

It is noteworthy that Notch-1-expressing AML cells were found in 90% of patients we examined in our study. However, the role of Notch-1 in AML progression still remains unclear. For instance, when GSI is administered to AML patients, the efficacy of this drug is minimal, indicating that Notch-1 is not a major player in driving AML clones. Recently, it has been shown that Notch mediates growth arrest and apoptosis in AML cells via activation of Hairy/Enhancer of Split 1 (HES1), suggesting the development of Notch agonists as a new therapy [24].

As we have previously demonstrated, L-fucose-mediated cell-targeting is a promising new therapeutic strategy for cancer treatment [11]. Herein, we show that the expression of CD33 and Notch-1 correlate in the same leukemia cell lines, and that Notch-1 positive leukemia cells are targeted by L-fucose-bound liposomes, Fuc-Liposome-daunorubicin particles were found to inhibit the growth of Notch-1 positive cells *in vitro* and in a mouse xenograft model, and suppressed Notch-1 positive, but not Notch-1 negative, AML cells derived from patients. Our results suggest that fucose-targeting therapy could be effective in increasing the survival of patients with AML.

## MATERIALS AND METHODS

### Materials

Daunorubicin was purchased from Santa Cruz (Dallas, TX, USA). Dipalmitoylphosphatidylcholine (DPPC), cholesterol (Chol), dicetyl phosphate (DCP), sodium cholate hydrate (cholic acid), human serum albumin (HSA), sodium periodate, deuterium oxide (D_2_O), sodium hexachloroplatinate, tris(hydroxymethyl)aminomethane (Tris), and L-fucose were purchased from Sigma (St. Louis, MO, USA). Ganglioside was purchased from Avanti Polar Lipids (Alabaster, AL, USA). Dipalmitoylphosphatidylethanolamine (DPPE) was purchased from Alexis (Plymouth Meeting, PA, USA). N-tris(hydroxymethyl)methyl-3-amino-propane sulfonic acid (TAPS) and n-(2-hydroxyethyl) piperazine-n'-(2-ethanesulfonic) acid were purchased from Doujin Chemical (Kumamoto, Japan). Sodium cyanoborohydrate was purchased from Aldrich (Milwaukee, WI, USA). BS^3^ and 3,3-dithiobis(sulfosuccinimidyl propionate) (DTSSP) were purchased from Pierce Biotechnology (Rockford, IL, USA). Cholesterol E-test Wako was purchased from Wako (Osaka, Japan).

### Patient cells and cell lines

Acute leukemia cells were obtained from patients after informed consent was obtained in accordance with the Declaration of Helsinki and institutional ethics committee. HL60, RPMI8226, KG1 and MOLT4 cells were obtained from the RIKEN Bioresource center (Tsubuka, Japan). The cells were cultured in RPMI 1640 (Life Technologies, Carlsbad, CA, USA) supplemented with 10% FBS, L-glutamine and 1% penicillin-streptomycin. These cells were authenticated by cytogenetics in 2013.

### Reverse transcription-polymerase chain reaction (RT-PCR)

Total RNA (1 μg) was reverse transcribed with a Superscript III first strand synthesis kit (Life Technologies) using random hexamers (100 pM) according to the manufacturer's instructions. cDNAs were amplified for 25 to 30 cycles using Pfu Turbo (Stratagene, La Jolla, CA, USA), 0.2 mM of each dNTP and 100 pM each primer. Each cycle consisted of 30 sec at 95°C, 30 sec at 55°C and 60 sec at 72°C. The sequences targeting human *POFUT* were as follows: POFUT: forward, 5′- TGA AGG AAG GAA ACC CCT TT -3′, reverse, 5′- TCT CCC GTC TTC ACC ATT TC -3′: internal standard ß-actin: forward, 5′-GCT CGT CGT CGA CAACGG CTC- 3′; reverse, 5′-CAA ACA TGC TCT GGG TCA TCT TCT- 3′.

### Preparation of FAM and daunorubicin encapsulated in liposomes

Preparation of FAM carrying liposomes has been described previously [11]. Briefly, DPPC, Chol, ganglioside, DCP, and DPPE were mixed at different molar ratios, and cholic acid added to facilitate micelle formation. The mixture was dissolved in methanol/chloroform (1:1, v/v), and the solvent was evaporated at 37°C to produce a lipid film, which was dried under vacuum. For the daunorubicin preparation, a HSA solution (HSA 20 mg/mL, 0.2 N NaOH) containing daunorubicin (20 mg/mL) was added to the lipid film and sonicated to obtain uniform micelles, which were then ultrafiltered (molecular weight cutoff 10 kD; Amicon PM10, Millipore, Billerica, MA, USA). Hydrophilization treatment and L-fucose conjugation on the surface of liposomes were carried out by methods modified from Yamazaki et al. [29]. Aminated L-fucose was conjugated to the liposome surface using DTSSP as described previously.

### Physicochemical characterization of Fuc-Liposome-daunorubicin

The average particle size and zeta-potential of liposomes were measured as described previously [11]. The lipid concentrations of Liposome-daunorubicin and Fuc-Liposome-daunorubicin were measured using a Cholesterol E-test Wako Kit as described previously [11].

### Measurement of daunorubicin and calculation of daunorubicin concentration and encapsulation efficiency

The concentration of daunorubicin was measured by absorbance at 485 nm. In brief, Fuc-Liposome-daunorubicin was diluted 1,000-fold with a methanol:chloroform (1:1) solution, and the concentration of daunorubicin was determined by absorbance at 485 nm. A calibration curve, with daunorubicin concentrations in the range of 625 – 5000 ng/mL was run before the analysis of each sample type.

Encapsulation efficiency (%) = (Amount of daunorubicin in liposomes/Initial amount of daunorubicin) × 100

Daunorubicin to lipid weight ratio = Daunorubicin concentration (mg/mL)/Lipid concentration (mg/mL)

### Flow cytometry analysis

Cells (1 × 10^5^) were incubated with Fuc-Liposome-FAM or Liposome-FAM (lipid concentration: 4 μg/mL) for 2 h. For the blocking assay, 1 × 10^5^ cells were treated with L-fucose for 30 min prior to adding Fuc-Liposome-FAM or Liposome-FAM. The mean fluorescence intensity (MFI) was assessed on a FACScalibur with CellQuest software (Becton Dickinson, Franklin Lakes, NJ, USA). For the detection of CD33 and Notch-1 expression, cell line samples (5 × 10^5^ cells each) were incubated with isotypic control, allophycocyanin (APC)-conjugated anti-Notch-1 antibody (R & D systems, Minneapolis, MN, USA) or FITC-conjugated anti-CD33 antibody (Miltenyl Biotec Bergisch Gladbach, Germany) on ice for 30 min. After washing in PBS/0.05% BSA, the cells were analyzed by flow cytometry (Beckton Dickinson).

### Intracellular distribution analysis of FAM

Cells were plated in Lab-Tek chambered coverglasses at 1 × 10^4^ cells/chamber. Fuc-Liposome-FAM or Liposome-FAM was added to cells at a final FAM concentration of 4 ng/ml. Cells were cultured in complete medium for 30 min, after which the medium was replaced with fresh medium. At 30 min and 2 h post-treatment, cells were washed twice with PBS and fixed with 4% paraformaldehyde for 15 min at room temperature. After fixation, cells were washed three times with PBS and exposed to Prolong Gold Antifade Reagent with DAPI (Life Technologies) for 10 min to stain nuclei. The subcellular localization of FAM was assessed by fluorescence microscopy (BZ-8000; Keyence, Osaka, Japan).

### Cell proliferation assay

Human leukemia cell lines (2 × 10^4^ cells/well) were seeded into 24-well plates, and cultured for one day in medium supplemented with 10% fetal bovine serum, 5% L-glutamine, and 1% antibiotics. Cells were then incubated with different doses of Fuc-Liposome-daunorubicin. After 2 h incubation, cells were washed twice with PBS and finally resuspended in medium containing serum and antibiotics. After culture for 72 h, a BrdU cell proliferation assay reagent (Cell Signaling, Boston, MA, USA) was added and the assay performed according to manufacturer's instructions. Experiments were performed in triplicate and repeated at least twice.

### AML xenograft model and treatment schedule

In a subcutaneous model, 2 × 10^6^HL60 cells were inoculated to create a dorsal lesion (mice aged 4 to 6 weeks) and each was allowed to grow into a tumor 5 mm in diameter. Tumor-bearing mice were randomly assigned to one of the following groups (n = 6 mice per group): no treatment, daunorubicin (1 mg/kg), F0-Liposome-daunorubicin (1 mg/kg), and F50-Liposome-daunorubicin (1 mg/kg). Treatments were administered through tail vein injection with a single dose of 100 μl of the above-mentioned drugs, twice a week for three weeks. At 4, 8, 11, 15, 18, 22 and 25 days after transplantation, tumor volumes were measured. For D-mannose pre-treatment in all *in vivo* experiments, 5 mg of D-mannose was injected through a tail vein 5 min prior to the administration of agents as described previously [11].

These studies were carried out according to the Guide for the Care and Use of Laboratory Animals of the National Institutes of the Health. The protocols were approved by the Committee on the Ethics of Animal Experiments of Sapporo Medical University. All surgery was performed under sodium pentobarbital anesthesia, and all efforts were made to minimize suffering. All mice underwent euthanasia when tumor volumes reached 20 mm in diameter.

### Statistics

Results are presented as mean ± standard deviation (SD) for each sample. Differences between the two groups were examined by unpaired and paired *t* tests. If two groups could not be considered to be of equal variance, a *t* test with Welch's correction was performed. The correlation analysis was tested by Pearson's product-moment correlation coefficient. All statistical analyses were performed using JMP, version 5.0 for Windows (SAS, Cary, NC, USA). Results were considered statistically significant when *P* < 0.05.

## SUPPLEMENTARY DATA FIGURES AND TABLES


