# Supplementary material for: Targeting Notch-1 positive acute leukemia cells by novel fucose-bound liposomes carrying daunorubicin
Source: Oncotarget. 2016 May 23;7(25):38586–97. doi: 10.18632/oncotarget.9558 (PMC5122413; doi:10.18632/oncotarget.9558)
Supplement: Supplementary file 1 [file oncotarget-07-38586-s001.pdf]

Targeting Notch-1 positive acute leukemia cells by novel fucose-bound liposomes carrying daunorubicin

SUPPLEMENTARY DATA

Reverse transcription-polymerase chain reaction (RT-PCR)

Total RNA (1 µg) was reverse transcribed with a Superscript III first strand synthesis kit (Life Technologies) using random hexamers (100 pM) according to the manufacturer's instructions. cDNAs were amplified for 25 to 30 cycles using Pfu Turbo (Stratagene), 0.2 mM of each dNTP and 100 pM each primer. Each cycle consisted of 30 sec at 95°C, 30 sec at 55°C and 60 sec at 72°C. The sequences targeting the human *FUTs* are as follows: *FUT1*; sense, 5'- ATG TGG CTC CGG AGC CAT CGT CAG -3', antisense, 5'- AGG ATC TCT CAA GTC CGC GTA CTC -3', *FUT2*; sense, 5'- CTA GCG AAG ATT CAA GCC ATG TGG -3', antisense, 5'- GAC GTA CTC CCC CGG GAT GTG -3', *FUT3*; sense, 5'- ATG GAT CCC CTG GGT GCA GCC AAG -3', antisense, TCA GGT GAA CCA AGC CGC TAT GCT -3', *FUT4*; sense, 5'- GTG CCC GAA ATT GGG CTC CTG CAC -3', antisense, 5'- GAA GGA GGT GAT GTG GAC AGC GTA -3', *FUT5*; sense, 5'- CTT ATG GCA GTG GAA CCT GTC ACC -3', antisense, 5'- CCA GCC GTA GGG CGT GAA GAT GTC -3', *FUT6*; sense, 5'- CCC ACT GTG TAC CCT AAT GGG TCC -3', antisense, 5'- CTC TCA GGT GAA CCA AGC CGC TAT -3', *FUT7*; sense, 5'- TCG GAC ATC TTT GTG CCC TAT G -3', antisense, 5'- CGC CAG AAT TTC TCC GTA ATG TA -3', *FUT*8; sense, 5'- TGC CTG GGG GAC CTT GCT GT -3', antisense, 5'- CCC GCC AAT CAC CTG CTC CA-3', *FUT9*; sense, 5'- CTT ACC GCC GTG ATT CAG AT -3', antisense, 5'- AAT GCT TGC CCG TAG GTA TG -3', *FUT10*; 5'- CTA TGT TCG CGA GCT GAT GA -3', antisense, 5'- TTT CAG TGG CCT CCA GAA CT -3'.

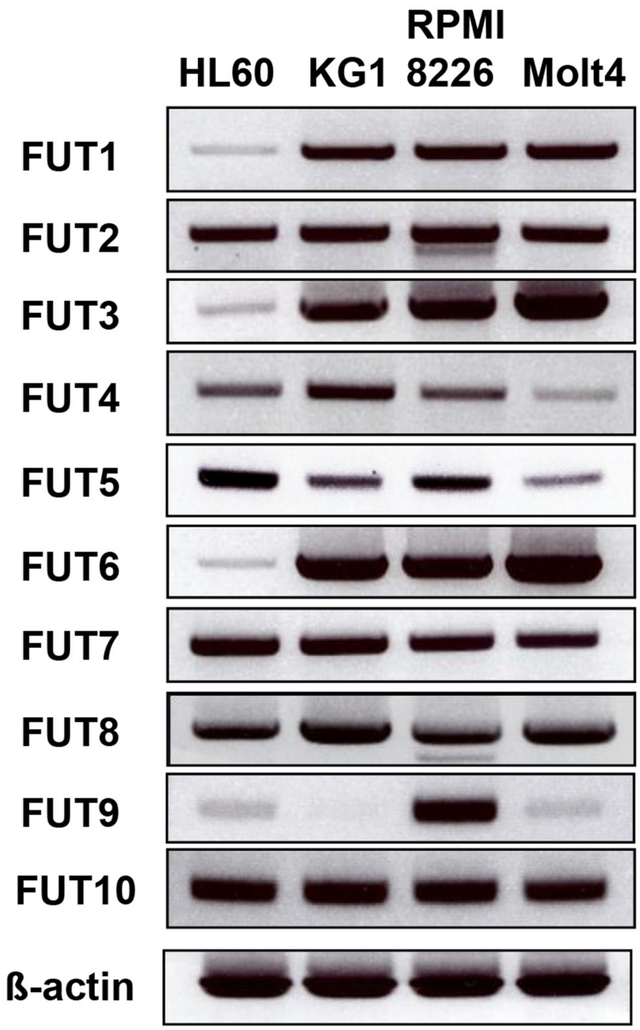

Supplementary Figure S1: Expression of FUTs in leukemia cell lines. Expression of FUTs mRNA in leukemia cells lines examined by semi-quantitative PCR. These experiments were performed three times.

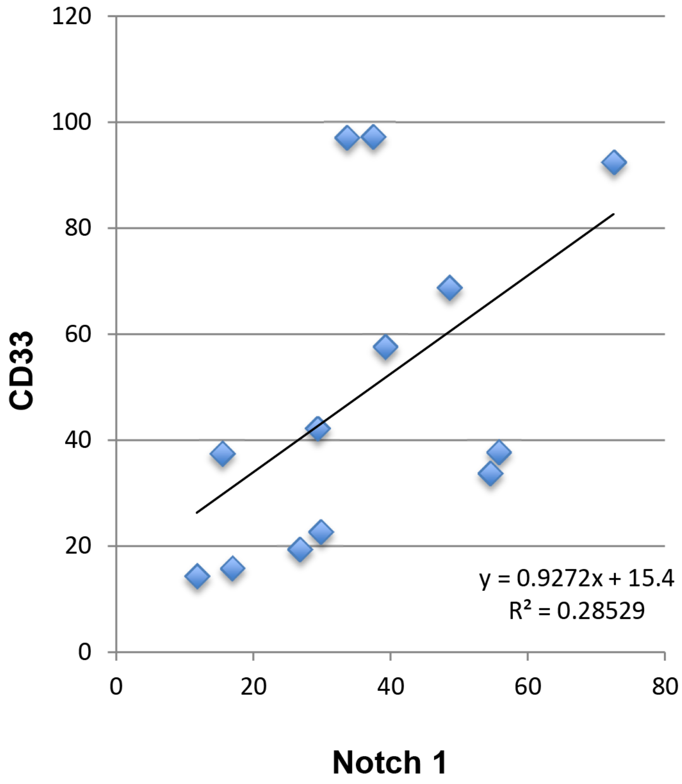

Supplementary Figure S2: Correlation analysis between CD33 and Notch1. Expression of CD33 and Notch1 was analysed by flow cytometry, and Pearson's correlateon analysis was carried out.

Supplementary Table S1: Physicochemical characteristics of FAM liposomes

Fucose concentration in conjugating reaction (μg/mL)	0 (F0)	25 (F25)	50 (F50)
Lipid concentration (mg/mL)[a]	**3.4**	**3.8**	**3.7**
Particle size (nm)	**73**	**98**	**72**
ζ potential (mV)[b]	**-64**	**-43**	**-45**
protein concentration (mg/mL)[c]	**0.7**	**0.7**	**0.8**
protein to lipid weight ratio[d]	**0.21**	**0.18**	**0.22**

(FAM-Lip and FAM-Fuc-Lip were concentrated 10-fold.)

[a]Total cholesterol was measured using a Cholesterol E-test Wako Kit.
[b]Determined with a Malvern Nano-S90.
[c]The amount of protein was measured based on OD at 680 nm.
[d]Protein: lipid weight ratio was calculated by the following formula: protein concentration (mg/mL)/lipid concentration (mg/mL).

Supplementary Table S2: Physicochemical characteristics of daunorubicin liposomes

Fucose concentration in conjugating reaction (μg/mL)	0 (F0)	25 (F25)	50 (F50)
Lipid concentration (mg/mL)[a]	**12.4**	**15.2**	**15.1**
Particle size (nm)[b]	**173**	**170**	**171**
ζ potential (mV)[c]	**-66**	**-62**	**-64**
daunorubicin concentration (mg/mL)[d]	**1.0**	**1.0**	**1.0**
daunorubicin encapsulation efficiency (%)[e]	**5.0**	**5.0**	**5.0**
daunomycin to lipid weight ratio[f]	**0.081**	**0.066**	**0.066**

[a]Total cholesterol was measured using a Cholesterol E-test Wako Kit.
[b]Encapsulation of daunorubicin into liposomes followed by conversion in TAPS buffer containing NaCl
[c]Determined with a Malvern Nano-S90.
[d]The amount of daunorubicin was measured by atomic absorption spectrophotometer.
[e]Encapsulation efficiency was calculated by the following formula: Amount of daunorubicin in liposomes/Initial amount of chemicals) × 100
[F]Daunorubicin:lipid weight ratio was calculated by following formula: daunorubicin concentration (mg/mL)/lipid concentration (mg/mL).
